# Gradient HPLC Method for Simultaneous Determination of Eight Sartan and Statin Drugs in Their Pure and Dosage Forms

**DOI:** 10.3390/ph13020032

**Published:** 2020-02-20

**Authors:** Mohsen M. Zareh, Monir Z. Saad, Wafaa S. Hassan, Mostafa E. Elhennawy, Mahmoud K. Soltan, Moustafa M. Sebaiy

**Affiliations:** 1 Department of Chemistry, Faculty of Science, Zagazig University, Zagazig 44519, Egypt; mohsenzareh@hotmail.com (M.M.Z.); zabdelsamii@gmail.com (M.Z.S.); mostafahenawy1@gmail.com (M.E.E.); 2 Department of Analytical Chemistry, Faculty of Pharmacy, Zagazig University, Zagazig 44519, Egypt; bellal_elmadina@yahoo.com; 3 Department of Medicinal Chemistry, Faculty of Pharmacy, Zagazig University, Zagazig 44519, Egypt; 4 Oman College of Health Sciences, Ministry of Health, Muscat 123, Sultanate of Oman; 5 Department of mathematical and physical sciences, University of Chester, Chester CH2 4NU 01244, UK

**Keywords:** gradient, HPLC, sartan, statin, dosage forms

## Abstract

A gradient HPLC method was developed and validated for rapid simultaneous separation and determination of the following eight drugs of sartan and statin classes in their pure and dosage forms within 15 minutes: irbesartan (IRB), losartan (LOS), valsartan (VAL), olmesartan (OLM), rosuvastatin (ROS), atorvastatin (ATR), lovastatin (LOV), and simvastatin (SIM). Separation was carried out on a Kinetex C_18_ 100A column (2.60 μm, 4.60 mm × 100 mm) using a gradiant binary mobile phase of 0.05M potassium dihydrogen phosphate buffer (pH 3.50 adjusted by ortho-phosphoric acid) and acetonitrile at room temperature. The flow rate was 1.00 mL/min and maximum absorption was measured using a DAD detector at 280 nm. The retention times of IRB, LOS, ROS, VAL, ATR, LOV, OLM, and SIM were recorded to be 4.72, 5.32, 6.06, 7.19, 7.96, 9.30, 11.91, and 14.66 minutes, respectively. Limits of detection were reported to be 2.01, 1.32, 1.10, 0.76, 0.21, 1.50, 0.38, and 0.55 mM for the same sequence of drugs, respectively, showing a high degree of method sensitivity. The method was then validated according to the international conference of harmonization (ICH) guidelines for the determination of the drugs in their dosage forms with highly precise recoveries. Also, a statistical comparison with reference methods was performed showing no significant differences between the proposed method and reported ones in terms of precision and accuracy.

## 1. Introduction

Blood pressure is the measure of the force of blood pushing against blood vessel walls. The heart pumps blood into blood vessels, which carry the blood throughout the body. High blood pressure, also called hypertension, is dangerous and fatal because it makes the heart work harder to pump blood out to the body and contributes to hardening of the arteries (atherosclerosis), stroke, kidney disease, and heart failure [1].

**Figure 1:**
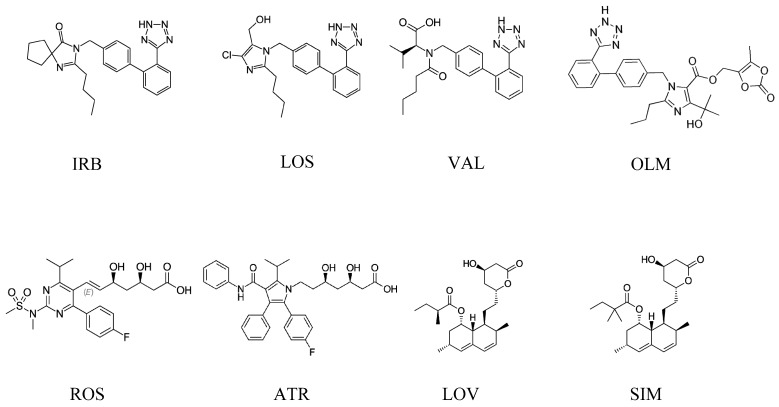
Chemical structures of irbesartan (IRB), losartan (LOS), valsartan (VAL), olmesartan (OLM), rosuvastatin (ROS), atorvastatin (ATR), lovastatin (LOV), and simvastatin (SIM).

Irbesartan (IRB) (2-butyl-3-{[2’-(1H-tetrazol-5-yl)biphenyl-4-yl]methyl}-1,3-diazaspiro[4.4]non-1-en-4-one) [2], losartan (LOS) ([2-butyl-5-chloro-3-[[4-[2-(2H-tetrazol-5-yl)phenyl]phenyl]methyl]imidazol-4-yl]methanol) [3], valsartan (VAL) (2S)-3-methyl-2-[pentanoyl-[[4-[2-(2H-tetrazol-5-yl)phenyl]phenyl]methyl]amino]butanoic acid [4], and olmesartan (OLM) (5-(2-hydroxypropan-2-yl)-2-propyl-3-[[4-[2-(2H-tetrazol-5-yl)phenyl]phenyl]methyl]imidazole-4-carboxylic acid) [5] are related to the sartan class (Figure 1), which is used to treat hypertension and to help protect the kidneys from damage due to diabetes. Sartans are angiotensin II receptor blockers (ARBs), also known as angiotensin II receptor antagonists, that modulate the renin–angiotensin system resulting in a decrease of unusual high blood pressure.

On the other hand, statins (Figure 1) are a class of drugs often prescribed by doctors to help lower cholesterol levels in the blood. By lowering cholesterol levels, they also help prevent heart attacks and stroke. Studies show that, in certain people, statins reduce the risk of heart attack, stroke, and even death from heart disease by about 25%–35%. Studies also show that statins can reduce the chances of recurrent strokes or heart attacks by about 40%. Statins used in this research were rosuvastatin (ROS) (E,3R,5S)-7-[4-(4-fluorophenyl)-2-[methyl(methylsulfonyl)amino]-6-propan-2-ylpyrimidin-5-yl]-3,5-dihydroxyhept-6-enoic acid [6], atorvastatin (ATR) (3R,5R)-7-[2-(4-fluorophenyl)-3-phenyl-4-(phenylcarbamoyl)-5-propan-2-ylpyrrol-1-yl]-3,5-dihydroxyheptanoic acid [7], lovastatin (LOV) [(1S,3R,7S,8S,8aR)-8-[2-[(2R,4R)-4-hydroxy-6-oxooxan-2-yl]ethyl]-3,7-dimethyl-1,2,3,7,8,8a hexahydronaphthalen-1-yl] (2S)-2-methylbutanoate [8], and simvastatin (SIM) [(1S,3R,7S,8S,8aR)-8-[2-[(2R,4R)-4-hydroxy-6-oxooxan-2-yl]ethyl]-3,7-dimethyl-1,2,3,7,8,8a-hexahydronaphthalen-1-yl]2,2-dimethyl butanoate [9]. Usually, combinations of statins and sartans are prescribed all over the world for patients with chronic heart failure, strokes, and ischemia. 

Due to the importance of these drugs in treating such fatal diseases, it is recommended to continuously develop new analytical methods to check purity and determine their potency. To the best of our knowledge through comprehensive survey, IRB, LOS, ROS, VAL, ATR, LOV, OLM, and SIM have been analyzed using chromatographic methods either alone or in combination with other related drugs [2,3,4,5,6,7,8,9,10,11,12,13,14,15,16,17,18], but these mixtures have not been determined in pharmaceutical nor in biological samples despite the importance of such separation to avoid the manipulation or adulteration that could happen from drug suppliers. As such, the present work introduces a simple, rapid, reproducible and sensitive chromatographic method for the determination of the cited drugs in their pure and dosage forms.

## 2. Experimental

### 2.1. Apparatus

We used an **Agilent 1100^®^** HPLC instrument (Waldbronn, Germany) with a Kinetex C_18_ 100A column (2.60 μ, 4.60 mm × 100 mm) (Aschaffenburg, Germany), DAD absorbance detector (Waldbronn, Germany), and HPLC QUAT pumps (Waldbronn, Germany) connected to a PC computer loaded with Agilent 1100 software. **Jenway^®^** 6800 Spectro UV-VIS Double Beam Spectrophotometer (Chelmsford, UK) with matched 1 cm quartz cells and connected to a windows compatible computer loaded with Flight Deck Software was also used. We also used a **HANNA^®^** HI 8314 membrane pH-meter (Cluj, Romania) for pH adjustment.

### 2.2. Materials and Reagents

All solvents and reagents were of an HPLC analytical grade (acetonitrile, potassium dihydrogen phosphate, and ortho-phosphoric acid were provided by Fisher Scientific, England). **IRB, LOS, ROS, VAL, ATR, LOV, OLM, and SIM** were kindly provided by different Egyptian companies such as Egyptian Company for Pharmaceutical & Chemical Industries (EIPICO), Egyphar Company, Delta Pharm Company, and Multi Apex Pharma, with purity ranging from 98% to 99.5%. Standard solutions were prepared by dissolving 20 mg of each pure drug in 100 mL of the mobile phase (50–50). **Mobile phase** consisted of two phases: (**A**) 0.05M potassium dihydrogen phosphate buffer (pH 3.50 by ortho-phosphoric acid) and (**B**) acetonitrile, filtered and degassed using a 0.45µm membrane filter. The gradient system ran for 20 minutes: mobile phase A was 90 →10, mobile phase B was 10 →90.

### 2.3. Pharmaceutical Formulations

Irbesartan^®^ (150mg IRB, Pfizer, Dokki city, Egypt), Losazide^®^ (50mg LOS, EIPICO, 10th of Ramadan City, Egypt), Estromap^®^ (20mg ROS, MULTI-APEX, Badr city, Egypt), Tareg^®^ (80mg VAL, NOVARTIS, Nasr city, Egypt), Ator^®^ (10mg ATR, EIPICO, Egypt), Lovastmed^®^ (40mg LOV, MASH PHARMA, Fifth Settlement city, Egypt), Erastapex^®^ (40mg OLM, APEX PHARMA, New Cairo city, Egypt), and Alkor^®^ (20mg SIM, HIKMA PHARMA, 6th of October city, Egypt) were purchased and subjected to analysis.

### 2.4. Procedures

#### 2.4.1. Preparation of Standard Calibration Curves

Appropriate mixed dilutions of IRB, LOS, ROS, VAL, ATR, LOV, OLM, and SIM standard stock solutions were prepared in 10 mL volumetric flasks to give 5 final concentrations. Then, 10 μL of each mixture was injected into the column and the chromatogram was obtained at 280 nm. A graph was plotted as concentration of drugs against response (peak area). Regarding validation quality control (QC) samples, concentrations were selected as low (LQC), medium (MQC), and high (HQC) levels.

#### 2.4.2. Pharmaceutical Dosages Procedure

Five tablets of Irbesartan^®^, Losazide^®^, Estromap^®^, Tareg^®^, Ator^®^, Lovastmed^®^, Erastapex^®^, and Alkor^®^ were weighed and powdered. Amounts equivalent to 20 mg of each drug were dissolved in the mobile phase, filtered through Whatman filter paper (Merck, Darmstadt, Germany) into 100 mL measuring flasks, and completed to volume with the mobile phase. The procedure was then completed as mentioned above under the general procedure 2.4.1, applying the standard addition technique.

## 3. Results and Discussion

### 3.1. Optimization of Chromatographic Conditions

**Figure 2:**
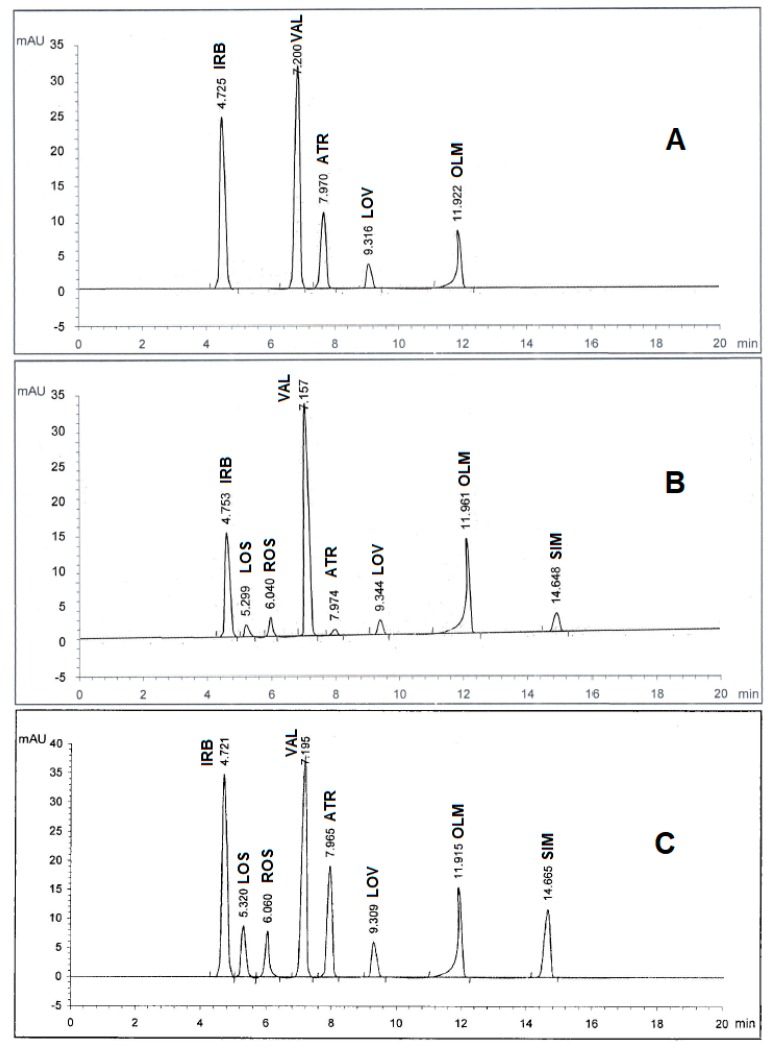
HPLC chromatogram of authentic mixture containing irbesartan (IRB), losartan (LOS), rosuvastatin (ROS), valsartan (VAL), atorvastatin (ATR), lovastatin (LOV), olmesartan (OLM), and simvastatin (SIM) using a Kinetex C_18_ 100A column (2.60 μm, 4.60 mm × 100 mm) and a gradient mobile phase 0.05M potassium dihydrogen phosphate buffer (pH 3.50 adjusted by ortho-phosphoric acid) and acetonitrile at different wavelengths: (**A**) 230 nm, (**B**) 254 nm, and (**C**) 280 nm. Other chromatographic conditions are stated in Table 1.

Spectroscopic analysis of the eight drugs in the range of 200–400 nm showed that they have diverse maximum wavelengths. As such, it was necessary to undergo an additional full scan on the Agilent 1100 software to determine which wavelength range is the most appropriate for the simultaneous determination of the drug mixture. As depicted in Figure 2, three wavelength ranges of 230, 254, and 280 nm were used for the trial scans and it was found that 280 nm achieved the best area under peak appearance and value for the eight drugs. Therefore, the chromatographic detection was performed at 280 nm as the appropriate wavelength using the DAD detector. The experiment was performed on a Kinetex C_18_ 100A column (2.60 μ, 4.60 mm × 100 mm). Furthermore, under several trials of mobile phase optimization regarding composition ratio and pH, it was observed that the optimized mobile phase was determined as a gradient mixture of (A) 0.05M potassium dihydrogen phosphate buffer (pH 3.50 adjusted by ortho-phosphoric acid) and (B) acetonitrile within 20 minutes in the following sequence: 0 minutes (A90:B10), 5 minutes (A50:B50), and 10 minutes (A10:B90) at ambient temperature with a flow rate 1 mL/min. Under these conditions, IRB, LOS, ROS, VAL, ATR, LOV, OLM, and SIM were recorded to be 4.72, 5.32, 6.06, 7.19, 7.96, 9.30, 11.91, and 14.66 minutes, respectively, as illustrated in Figure 2C. However, in all cases, the optimum mobile phase showed symmetrical peaks (0.80 < T < 1.20), capacity factor < 10, resolution > 2, and theoretical plates > 2000, which are in agreement with the Center for Drug Evaluation and Research (CDER) value recommendations [19]. All chromatographic conditions are illustrated in Table 1.

### 3.2. Method Validation

The method validation was performed according to international conference of harmonization guidelines (ICH) [20].

#### 3.2.1. Linearity

Linearity studies of five different concentrations of the drug mixtures were repeated three times. The calibration curves obtained by plotting peak area against concentration showed linearity in different concentration ranges as specified in Table 2. Linear regression equations of IRB, LOS, ROS, VAL, ATR, LOV, OLM, and SIM were found to be y = 6.9214× + 4.1449, y = 1.7537× + 0.6935, y = 1.5676× + 0.7195, y = 7.7221× + 2.761, y = 3.9397× + 1.4593, y = 1.2083× + 0.7765, y = 3.1378× + 1.3978, and y = 2.3767× + 1.1512, respectively, and the regression coefficient values (r) were calculated to be 0.9999 for IRB, and 1 for the remaining drugs, indicating a high degree of linearity.

#### 3.2.2. Accuracy

The accuracy of the method was determined by investigating the recoveries of commercial formulations at different 3 concentrations (three replicates) using the standard addition technique. It was performed by adding a fixed standard concentration for each drug at different levels and the proposed method was followed. From the amount of the drug estimated, the percentage recovery was calculated and the results are shown in Table 3 and indicate excellent recoveries for all drugs.

#### 3.2.3. Precision

The precision of the method was evaluated according to intra-day and inter-day precision using validation QC samples at concentrations as seen in Table 4. Intra-day precision was evaluated in respect to both standard deviation (SD) and coefficient of variation (CV%) regarding three replicate determinations using the same solution containing pure drugs. The SD values (0.15 to 1.22) and CV% values (0.15 to 1.23) in Table 4 revealed the high precision of the method. For inter-day reproducibility, the day-to-day SD and CV% values were also in the acceptable range of 0.38–1.22 and 0.39–1.21, respectively (Table 4). These results show that the proposed method has an adequate precision with respect to the simultaneous determination of the eight cited drugs in their pharmaceutical formulations.

#### 3.2.4. Selectivity and Specificity

**Figure 3:**
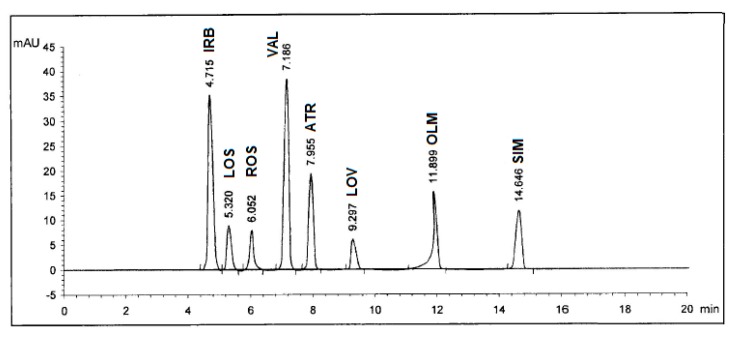
HPLC Chromatogram of authentic mixture containing Irbesartan^®^ (IRB), Losazide^®^ (LOS), Estromap^®^ (ROS), Tareg^®^ (VAL), Ator^®^ (ATR), Lovastmed^®^ (LOV), Erastapex^®^ (OLM), and Alkor^®^ (SIM) tablets dosage forms at 280 nm. Other optimum chromatographic conditions are stated in Table 1.

Selectivity of the method was checked by injecting the solutions of IRB, LOS, ROS, VAL, ATR, LOV, OLM, and SIM into the column separately where eight sharp peaks were obtained at retention times of 4.72, 5.32, 6.06, 7.19, 7.96, 9.30, 11.91, and 14.66 minutes, respectively, and these peaks were not obtained for blank solutions. Also, the specificity studies revealed that the presence of the excipents in the tablet formulations did not show any kind of impurity interference with the sharp and well-resolved peaks of the eight drugs (Figure 3). 

#### 3.2.5. Limits of Detection and Limits of Quantification

For determining the limits of detection (LOD) and limits of quantification (LOQ), the method based on signal-to-noise ratio (3:1 for LOD and 10:1 for LOQ) was adopted. Limits of detection were reported to be 2.01, 1.32, 1.10, 0.76, 0.21, 1.50, 0.38, and 0.55 mM, while limits of quantification were calculated to be 6.11, 4.02, 3.36, 2.27, 0.68, 4.57, 1.11, and 1.65 mM for IRB, LOS, ROS, VAL, ATR, LOV, OLM, and SIM, respectively (Table 2). These results show that the proposed method is highly sensitive and applicable not only for pharmaceutical analysis but also for pharmacokinetic studies.

#### 3.2.6. Robustness

The robustness of the method was evaluated by making deliberate subtle changes in the flow rate, mobile phase composition ratio, and temperature of samples keeping the other chromatographic conditions constant. The effects of the changes were studied on the basis of percent recovery and standard deviation of all drugs. Table 5 shows that the changes had negligible influence on the results as revealed by small SD values for all applied changes.

### 3.3. Analysis of Pharmaceutical Formulations

Irbesartan^®^, Losazide^®^, Estromap^®^, Tareg^®^, Ator^®^, Lovastmed^®^, Erastapex^®^, and Alkor^®^ pharmaceutical formulations containing IRB, LOS, ROS, VAL, ATR, LOV, OLM, and SIM, respectively, were successfully analyzed by the proposed method. Excipients and impurities did not show interference, indicating a high degree of specificity for the method. Results obtained were compared to those obtained by reference methods [2,3,4,5,6,7,8,9] using the Graph Pad Prism 5 program where student’s t-test and F-test were performed for comparison. Results shown in Table 6 indicated that calculated t and F values were less than the tabulated ones for the eight drugs, which in turn indicates that there is no significant difference between the proposed method and reference ones relative to precision and accuracy.

## 4. Conclusions

The presented method was developed and validated for rapid simultaneous estimation of sartan and statin drugs within 15 minutes. The results obtained indicate that the proposed method is rapid, accurate, selective, robust, and reproducible. Linearity was observed over a concentration range of 5–100 μg/mL for all drugs. The method was successfully applied for the analysis of marketed formulations with respect to quality control in addition to performing statistical comparisons with reference methods showing no significant differences.

## Figures and Tables

**Table 1:** Chromatographic conditions for the proposed method.

Parameters	Conditions
**Column**	Kinetex C_18_ 100A column (2.60 μm, 4.60 mm × 100 mm).
**Mobile Phase**	**A:** 0.05M potassium dihydrogen phosphate buffer (pH 3.50 by ortho-phosphoric acid) **B:** Acetonitrile Gradient system within 20 min Mobile phase A: 90 →10 Mobile phase B: 10 →90 at 0 min (A90:B10), at 5 min (A50:B50), at 10 min (A10:B90)
**UV Detection, nm**	280
**Flow Rate, mL/min**	1
**Injected Volume, µL**	10
**Temperature**	Ambient (25 ± 2)

**Table 2:** Analytical merits for determination of irbesartan (IRB), losartan (LOS), rosuvastatin (ROS), valsartan (VAL), atorvastatin (ATR), lovastatin (LOV), olmesartan (OLM), and simvastatin (SIM) in pure samples using the proposed method.

	**IRB**				**LOS**				**ROS**				**VAL**			
	**Conc. Taken (mM)**	**Conc. Found (mM)**	**Recovery**	**% Accuracy**	**Conc. Taken (mM)**	**Conc. Found (mM)**	**Recovery**	**% Accuracy**	**Conc. Taken (mM)**	**Conc. Found (mM)**	**Recovery**	**% Accuracy**	**Conc. Taken (mM)**	**Conc. Found (mM)**	**Recovery**	**% Accuracy**
	**11.67**	11.46	98.29	−1.70	**11.82**	11.61	98.15	−1.85	**10.38**	10.20	98.12	−1.88	**11.48**	11.23	97.79	−2.20
	**23.34**	22.78	97.66	−2.33	**23.65**	23.50	99.48	−0.51	**20.77**	20.52	98.88	−1.12	**22.96**	22.92	99.76	−0.23
	**46.67**	47.98	102.80	2.80	**47.29**	48.09	101.69	1.69	**41.53**	42.26	101.78	1.78	**45.92**	46.38	101.09	1.09
	**116.68**	115.93	99.36	−0.60	**118.23**	117.59	99.45	−0.54	**103.83**	103.42	99.62	−0.38	**144.80**	114.58	99.80	−0.19
	**233.36**	233.52	100.07	0.07	**236.46**	236.69	100.08	0.08	**207.67**	207.75	100.04	0.04	**229.61**	229.84	100.01	0.014

**Mean**			99.64	−0.35			99.77	−0.23			99.69	−0.31			99.69	-0.30
**SD**			2.008				1.28				1.38				1.19	
**CV (%)**			2.01				1.28				1.385				1.197	
**SE**			0.89				0.57				0.62				0.53	
**Variance**			4.03				1.64				1.90				1.42	
**Slope**			6.90				1.75				1.57				7.72	
**LOD (mM)**			2.01				1.32				1.10				0.76	
**LOQ (mM)**			6.11				4.02				3.36				2.27	

	**ATR**				**LOV**				**OLM**				**SIM**			
	**Conc. Taken (mM)**	**Conc. Found (mM)**	**Recovery**	**% Accuracy**	**Conc. Taken (mM)**	**Conc. Found (mM)**	**Recovery**	**% Accuracy**	**Conc. Taken (mM)**	**Conc. Found (mM)**	**Recovery**	**% Accuracy**	**Conc. Taken (mM)**	**Conc. Found (mM)**	**Recovery**	**% Accuracy**
	**8.95**	9.06	101.23	1.23	**12.36**	12.36	100.03	0.03	**8.95**	9.00	100.72	0.72	**11.95**	11.99	100.55	0.55
	**17.90**	17.85	99.68	−0.32	**24.72**	23.98	97.02	−2.97	**17.90**	17.71	98.96	−1.03	**23.89**	23.56	98.58	−1.42
	**35.80**	35.68	99.69	−0.31	**49.44**	49.74	100.60	0.61	**35.80**	36.00	100.55	0.55	**47.78**	47.99	100.45	0.45
	**89.50**	89.57	100.08	0.08	**123.60**	124.44	100.67	0.67	**89.51**	89.40	99.88	−0.11	**119.46**	119.59	100.12	0.12
	**179.01**	178.99	99.99	−0.01	**247.19**	246.80	99.84	−0.16	**179.02**	179.04	100.01	0.01	**238.91**	238.82	99.96	−0.03

**Mean**			100.13	0.13			99.63	−0.36			100.03	0.03			99.93	-0.07
**SD**			0.64				1.50				0.69				0.79	
**CV (%)**			0.63				1.509				0.69				0.79	
**SE**			0.28				0.67				0.31				0.35	
**Variance**			0.40				2.26				0.47				0.63	
**Slope**			3.94				1.21				3.14				2.37	
**LOD (mM)**			0.21				1.50				0.38				0.55	
**LOQ (mM)**			0.68				4.57				1.11				1.65	

**Table 3:** Application of standard addition technique for the determination of Irbesartan^®^ (IRB), Losazide^®^ (LOS), Estromap^®^ (ROS), Tareg^®^ (VAL), Ator^®^ (ATR), Lovastmed^®^ (LOV), Erastapex^®^ (OLM), and Alkor^®^ (SIM) tablets using the proposed method.

	Conc. (mM)	Found Conc. (mM)	Mean ± SD	CV %	Accuracy %
**IRB** (Irbesartan 150mg^®^)	11.67	11.64	100.76 ± 1.04	0.46	0.76
**(n = 3)**	23.24	23.45			
	116.68	118.89			
**LOS** (Losazide 50mg^®^)	11.82	11.63	100.58 ± 1.90	0.86	0.58
**(n = 3)**	23.65	24			
	118.23	120.36			
**ROS** (Estromap 20mg^®^)	10.38	10.32	101.05 ± 1.41	0.63	1.05
**(n = 3)**	20.77	21.04			
	103.83	106.22			
**VAL** (Tareg 80mg^®^)	11.48	11.25	101.00 ± 2.68	1.2	1.003
**(n = 3)**	22.96	23.37			
	144.8	118.41			
**ATR** (Ator 10mg ^®^)	8.95	8.79	101.06 ± 2.49	1.11	1.06
**(n = 3)**	17.9	18.35			
	89.5	91.74			
**LOV** (Lovastmed 40mg ^®^)	12.36	12.16	100.23 ± 2.08	0.92	0.23
**(n = 3)**	24.72	24.67			
	123.6	126.62			
**OLM** (Erastapex 40mg ^®^)	8.95	8.84	101.04 ± 1.95	0.87	1.04
**(n = 3)**	17.9	18.22			
	89.51	91.71			
**SIM** (Alkor 20mg ^®^)	11.95	12.02	101.70 ± 1.31	0.59	1.71
**(n = 3)**	23.89	24.23			
	119.46	123.16			

**Table 4:** Intra- and inter-day precision results of sartans and statins in pure samples using the proposed method.

		Intra-Day Runs (n = 3)		Inter-Day Runs (n = 3)	
Drugs	Concentrations (mM)	Mean Recovery ± SD	CV (%)	Mean Recovery ± SD	CV (%)
**IRB**	11.67	99.10 ± 0.15	0.15	98.30 ± 0.60	0.61
	23.24	100.32 ± 0.16	0.17	99.90 ± 0.51	0.52
	116.68	100.47 ± 0.18	0.19	99.79 ± 0.57	0.57
**LOS**	11.82	98.00 ± 0.78	0.79	98.10 ± 0.53	0.54
	23.65	101.09 ± 0.63	0.62	101.71 ± 0.52	0.51
	118.23	101.17 ± 0.56	0.56	100.12 ± 0.52	0.51
**ROS**	10.38	99.39 ± 1.22	1.23	98.84 ± 0.38	0.39
	20.77	100.80 ± 0.86	0.85	101.10 ± 0.56	0.55
	103.83	100.90 ± 0.71	0.71	100.40 ± 1.22	1.21
**VAL**	11.48	98.87 ± 0.65	0.66	97.70 ± 0.51	0.52
	22.96	101.40 ± 0.45	0.44	101.20 ± 0.51	0.5
	144.8	101.50 ± 0.15	0.15	101.10 ± 0.53	0.52
**ATR**	8.95	100.60 ± 0.53	0.52	100.60 ± 0.53	0.53
	17.9	101.40 ± 0.98	0.97	101.91 ± 0.53	0.52
	89.5	102.00 ± 0.41	0.41	101.91 ± 0.54	0.53
**LOV**	12.36	100.60 ± 0.36	0.35	100.00 ± 0.57	0.57
	24.72	100.20 ± 0.29	0.3	99.29 ± 0.50	0.51
	123.6	101.40 ± 0.45	0.44	101.20 ± 0.53	0.52
**OLM**	8.95	99.64 ± 0.46	0.46	98.54 ± 0.52	0.53
	17.9	101.40 ± 0.47	0.46	101.20 ± 0.53	0.52
	89.51	101.70 ± 0.21	0.2	101.20 ± 0.67	0.66
**SIM**	11.95	100.90 ± 0.44	0.44	99.96 ± 0.51	0.51
	23.89	101.10 ± 0.31	0.3	100.90 ± 0.53	0.52
	119.46	101.40 ± 0.52	0.51	101.30 ± 0.52	0.51

**Table 5:** Results of the robustness for the determination of irbesartan (IRB), losartan (LOS), rosuvastatin (ROS), valsartan (VAL), atorvastatin (ATR), lovastatin (LOV), olmesartan (OLM), and simvastatin (SIM) (40 mM) using the proposed method.

**Parameter**	**IRB**		**LOS**		**ROS**		**VAL**	
	**Mean Recovery ± SD**	**CV (%)**	**Mean Recovery ± SD**	**CV (%)**	**Mean Recovery ± SD**	**CV (%)**	**Mean Recovery ± SD**	**CV (%)**
**Flow Rate 0.90**	100.08 ± 2.90	2.90	100.35 ± 2.47	2.46	100.14 ± 2.30	2.31	100.26 ± 2.24	2.23
**Flow Rate 1.10**	100.02 ± 2.78	2.78	100.29 ± 2.35	2.34	100.20 ± 2.43	2.43	100.20 ± 2.12	2.12
**Mobile Phase (92-8)**	99.97 ± 2.68	2.68	100.25 ± 2.55	2.55	100.16 ± 2.34	2.33	100.16 ± 2.03	2.03
**Mobile Phase (88-12)**	99.86 ± 2.46	2.46	100.15 ± 2.03	2.03	100.05 ± 2.11	2.11	100.05 ± 1.82	1.82
**Temp. 35 °C**	99.90 ± 2.55	2.56	100.19 ± 2.13	2.13	100.10 ± 2.22	2.21	100.10 ± 1.90	1.91
**Temp. 30 °C**	99.84 ± 2.41	2.42	100.12 ± 1.99	1.98	100.03 ± 2.07	2.07	100.03 ± 1.78	1.78
**Parameter**	**ATR**		**LOV**		**OLM**		**SIM**	
	**Mean Recovery ± SD**	**CV (%)**	**Mean Recovery ± SD**	**CV (%)**	**Mean Recovery ± SD**	**CV (%)**	**Mean Recovery ± SD**	**CV (%)**
**Flow Rate 0.90**	100.69 ± 1.16	1.15	100.20 ± 2.29	2.29	100.60 ± 1.70	1.69	100.49 ± 1.70	1.70
**Flow Rate 1.10**	100.60 ± 1.05	1.04	100.14 ± 2.18	2.18	100.55 ± 1.59	1.58	100.44 ± 1.60	1.60
**Mobile Phase (92-8)**	100.59 ± 0.97	0.97	100.09 ± 2.11	2.11	100.50 ± 1.49	1.48	100.39 ± 1.50	1.50
**Mobile Phase (88-12)**	100.48 ± 0.79	0.79	99.99 ± 1.90	1.93	100.39 ± 1.28	1.27	100.29 ± 1.30	1.30
**Temp. 35 °C**	100.50 ± 0.87	0.86	100.04 ± 2.02	2.02	100.44 ± 1.37	1.37	100.08 ± 0.97	0.96
**Temp. 30 °C**	100.47 ± 0.77	0.76	99.97 ± 1.90	1.90	100.37 ± 1.24	1.24	100.26 ± 1.27	1.27

**Table 6:** Statistical analyses of results obtained by the proposed method applied on Irbesartan^®^ (IRB), Losazide^®^ (LOS), Estromap^®^ (ROS), Tareg^®^ (VAL), Ator^®^ (ATR), Lovastmed^®^ (LOV), Erastapex^®^ (OLM), and Alkor^®^ (SIM) tablets compared with reference methods.

	**Irbesartan^®^ (IRB)**		**Losazide^®^ (LOS)**		**Estromap^®^ (ROS)**		**Tareg^®^ (VAL)**	
	**Proposed** **Method**	**Reference Method** [2]	**Proposed Method**	**Reference Method** [3]	**Proposed Method**	**Reference Method** [6]	**Proposed Method**	**Reference Method** [4]
**N**	3	3	3	4	3	3	3	3
**Mean Recovery**	98.36	99.38	99.03	100.20	98.84	99.92	98.16	96.98
**SE**	0.36	0.32	0.71	0.21	0.63	0.52	0.59	2.42
**Variance**	0.39	0.31	1.51	0.18	1.18	0.83	1.05	17.67
**Student-t**	**2.08 (2.13) ^a^**		**1.77 (2.01) ^a^**		**1.31 (2.13) ^a^**		**0.47 (2.13) ^a^**	
**F-test**	**1.25 (19.00) ^b^**		**8.18 (9.55) ^b^**		**1.42 (19.00) ^b^**		**16.08 (19.00) ^b^**	
	**Ator^®^ (ATR)**		**Lovastmed^®^ (LOV)**		**Erastapex^®^ (OLM)**		**Alkor^®^ (SIM)**	
	**Proposed Method**	**Reference Method** [7]	**Proposed Method**	**Reference Method** [8]	**Proposed Method**	**Reference Method** [5]	**Proposed Method**	**Reference Method** [9]
**N**	3	3	3	3	3	3	3	3
**Mean Recovery**	99.81	99.97	100.20	100.30	99.34	99.54	102.10	101.00
**SE**	0.32	0.12	0.04	0.06	0.37	0.09	0.61	0.60
**Variance**	0.31	0.05	0.01	0.01	0.43	0.02	1.10	1.08
**Student-t**	**0.46 (2.13) ^a^**		**1.11 (2.13) ^a^**		**0.05 (2.13) ^a^**		**1.36 (2.13) ^a^**	
**F-test**	**6.61 (19.00) ^b^**		**2.39 (19.00) ^b^**		**15.67 (19.00) ^b^**		**1.02 (19.00) ^b^**	

^a^ and ^b^ are the theoretical student t-values and F-ratios at *p* = 0.05.
